# Imaging biomarkers of liver hypertrophy after portal vein embolization in patients with liver tumors: cholestasis, arterial hyperperfusion, and splenic response

**DOI:** 10.1007/s00261-026-05540-y

**Published:** 2026-05-12

**Authors:** Anne B. Beeskow, Karoline Rucker, Jakob Leonhardi, Aboelyazid Elkilany, Daniel Seehofer, Hans-Michael Tautenhahn, Thomas Berg, Florian van Bömmel, Holger Gößmann, Timm Denecke, Florian Struck, Sebastian Ebel

**Affiliations:** 1 Department of Diagnostic and Interventional Radiology, University Hospital Leipzig, Leipzig, Germany; 2 Department of Visceral, Transplantation, Thoracic and Vascular Surgery, University Hospital Leipzig, Leipzig, Germany; 3 Medical Clinic II, Division of Hepatology, University Hospital Leipzig, Leipzig, Germany; 4 Department of Anesthesiology and Intensive Care Medicine, University Hospital Leipzig, Leipzig, Germany

**Keywords:** Portal vein embolization (PVE), Future liver remnant (FLR), hypertrophy Imaging biomarkers Cholestasis Arterial hyperperfusion, Liver venous deprivation (LVD)

## Abstract

**Purpose:**

To identify imaging and clinical factors associated with future liver remnant (FLR) hypertrophy after portal vein embolization (PVE) prior to major hepatectomy.

**Materials and methods:**

This retrospective single-center study included 98 patients (mean age 61 ± 14 years, 56 female) with benign or malignant liver tumors who underwent PVE between 2019 and 2024 before planned major liver resection. 10 patients also received liver venous deprivation (LVD). FLR volumes were measured on cross-sectional imaging before and after PVE. Embolic agent, cholestasis, arterial perfusion patterns, signal intensities in MRI, spleen volume, and venous diameters were assessed.

**Results:**

Mean FLR growth was 35.6 ± 22.2% (628.9 ± 256.2 to 979.6 ± 313.8 ml (p < 0.001)). Patients undergoing combined LVD demonstrated significantly greater FLR hypertrophy (mean FLR growth 61.3 ± 23.1%, 422 ± 97 ml; p < 0.001) compared with PVE alone. Arterial hyperperfusion of the embolized liver lobe was found to be associated with increased FLR growth (p = 0.036). Isolated cholestasis confined to the embolized lobe was also associated with greater FLR hypertrophy (p = 0.038), whereas cholestasis involving the FLR was associated with impaired growth (p = 0.042). No significant difference in FLR hypertrophy was observed regarding the used embolic agent (p = 0.11). Splenic volume increased significantly following PVE (311.9 ± 146.5 mL after PVE vs. 271.3 ± 118.5 mL prior to PVE, p < 0.001).

**Conclusion:**

Imaging features such as arterial hyperperfusion and cholestasis of the embolized liver lobe, and postinterventional splenic enlargement are associated with larger FLR-growth. These findings may provide valuable early indicators of regenerative response and could help refine patient selection for PVE and hepatectomy, warranting further prospective evaluation.

**Graphical abstract:**

**Figure:**
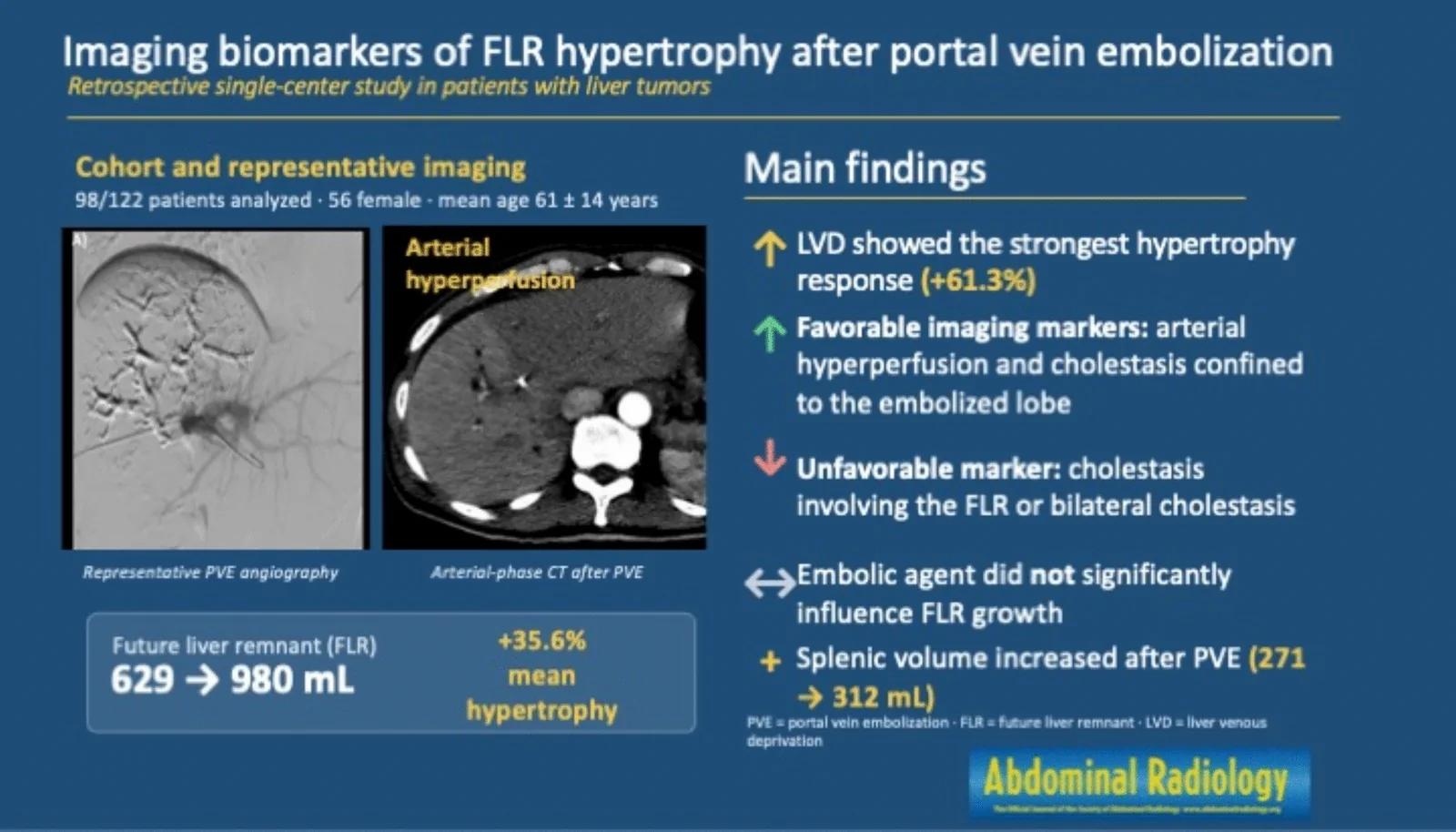

**Supplementary Information:**

The online version contains supplementary material available at https://doi.org/10.1007/s00261-026-05540-y.

## Introduction

Hemihepatectomy represents a potentially curative treatment option in cases of primary or secondary tumor involvement of a hepatic lobe. To reduce the risk of post-hepatectomy liver failure (PHLF) [1], portal vein embolization (PVE) of the diseased liver lobe is routinely performed 6–8 weeks prior to hepatectomy [2] in order to induce hypertrophy of the contralateral liver lobe, referred to as the future liver remnant (FLR). This approach is effective irrespective of tumor entity [3].

PVE is particularly relevant in right-sided hepatectomy, where hypertrophy of segments II and III is required, but it is also applicable in planned left-sided resections [4, 5]. The procedure is performed in a minimally invasive manner under image guidance. Both cyanoacrylate glue and particulate embolic agents are established materials for PVE [6, 7]. A higher degree of liver hypertrophy has been demonstrated with N-butyl cyanoacrylate compared with particulate embolization materials [7]. However, the choice of embolic agent largely depends on local expertise and institutional protocols.

To further enhance FLR hypertrophy, liver venous deprivation (LVD) has been proposed as an additional strategy [8–10].

Liver venous deprivation (LVD) performed in conjunction with portal vein embolization (PVE) has been reported to result in greater future liver remnant (FLR) hypertrophy compared with PVE alone, with higher rates of completion of planned hepatic resection and potentially lower incidence of post-hepatectomy liver failure. Meta-analytic data demonstrate that LVD yields significantly higher relative and daily FLR growth while maintaining a safety profile comparable to PVE in terms of procedure-related complications and 90-day mortality [11, 12]. Nevertheless, the current evidence base is predominantly retrospective, with substantial heterogeneity in embolization techniques, embolic agents, and timing of post-procedural imaging, limiting generalizability. Furthermore, some matched-pair analyses have found no statistically significant difference in FLR hypertrophy between LVD and PVE in non-cirrhotic patients, underscoring the need for prospective, randomized trials to establish the clinical benefit and standardized procedural protocols [13].

Major liver resection remains a high-risk procedure, even when performed after PVE with persistent risks of hepatic insufficiency, liver failure, and mortality [14]. In the worst case, it may result in death due to PHLF or insufficient FLR hypertrophy, thereby precluding further curative treatment of liver tumors. Morphological imaging markers, including MRI-based surrogates of liver function and steatosis (e.g., hepatobiliary contrast-enhanced MRI indices), have previously been investigated for predicting adequate FLR growth [15–17]. A smaller baseline future liver remnant (FLR) has consistently been associated with a greater hypertrophic response following portal vein embolization (PVE), demonstrating an inverse relationship between initial FLR volume and subsequent hypertrophy [18, 19]. In addition, higher intrahepatic tumor burden on imaging was independently and inversely correlated with FLR hypertrophy, irrespective of baseline volumetric measurements [20]. Functional imaging modalities, including technetium-mebrofenin hepatobiliary scintigraphy and contrast-enhanced MRI, have occasionally demonstrated regenerative capacity that surpasses volumetric increases, highlighting their potential utility in refining predictions of post-PVE hypertrophy [18].

Nevertheless, up to 20% of patients undergoing PVE remain ineligible for resection due to insufficient FLR hypertrophy [3]. Accurate preinterventional prediction of FLR hypertrophy therefore represents an unmet clinical need with direct implications for patient safety, eligibility for curative surgery, and overall survival. This study aims to evaluate the feasibility of additional imaging biomarkers for predicting FLR hypertrophy, with the goal of enabling personalized patient selection and improving outcomes after liver resection.

## Materials and methods

### Study design

In this retrospective, single-center study, all PVE procedures were performed by senior radiologists (> 8 years of experience). Approval from the institutional ethics committee was obtained, and written informed consent was provided by all patients. All methods were carried out in accordance with the relevant guidelines and regulations.

### Study cohort

**Fig. 1:**
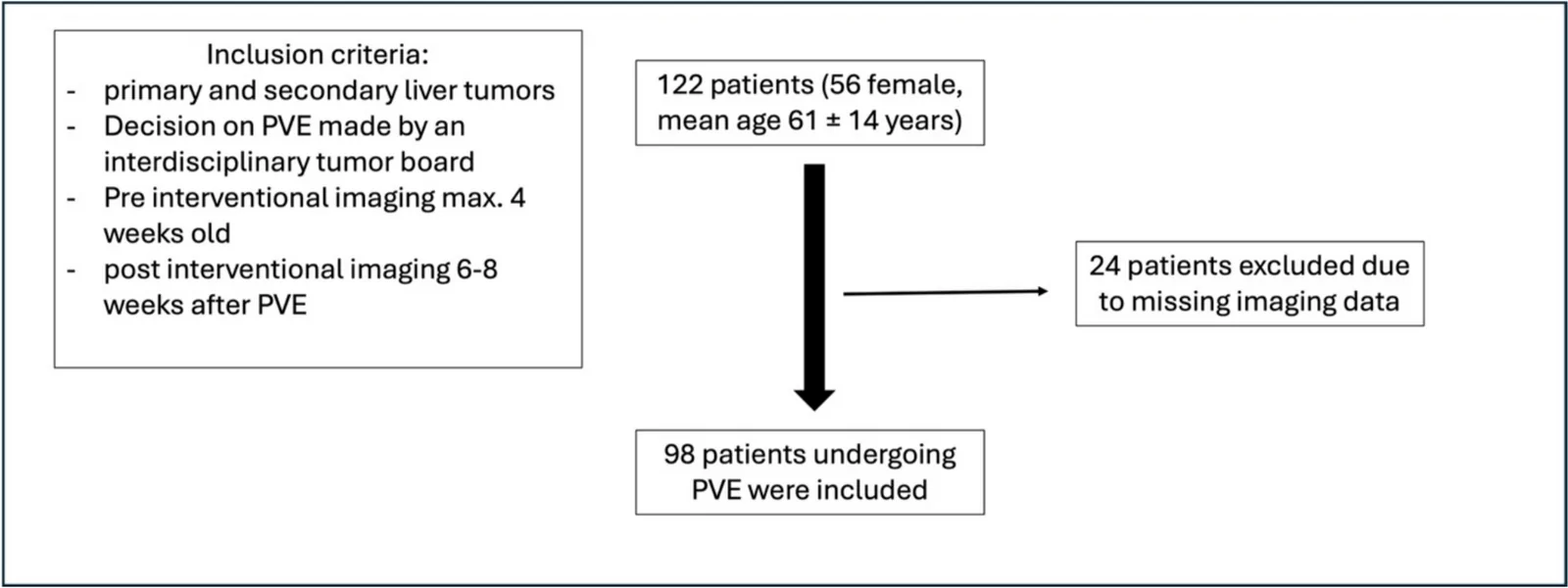
Flow Chart: inclusion criteria

A total of 122 patients (56 female; mean age 61 ± 14 years) with malignant (primary and secondary liver tumors) or benign liver tumors who underwent PVE between January 2019 and February 2024 were included in this retrospective analysis. The indication for PVE prior to resection was determined by an interdisciplinary tumor board. Twenty-four patients were excluded due to missing CT data (Fig. 1).

Based on recent publications [6], patients received additional right-sided liver venous deprivation (LVD) starting in August 2023. All LVD procedures were performed simultaneously with PVE (within the same intervention); no sequential LVD was performed. The present analysis was performed after inclusion of the first 10 patients treated with LVD. 22 patients had to be excluded due to missing imaging data. Ultimately, 98 patients (53 female; mean age 62 ± 12 years) were included in the final analysis. The mean body mass index was 26.4 ± 5.4 kg/m^2^. The cohort comprised 53 patients with cholangiocellular carcinoma (CCC), 5 with hepatocellular carcinoma (HCC), 31 with liver metastases, and 9 with benign liver lesions (Table 1). All patients underwent FibroScan vibration-controlled transient elastography [21], with the following parameters assessed: controlled attenuation parameter (CAP) and liver stiffness measurement (LSM). A CAP value (dB/m) ≥ 1 was considered indicative of steatosis. A LSM value (kPa) ≥ 3 was classified as advanced fibrosis, and an LSM value of 4 was considered cirrhosis. The stages of cirrhosis were classified according to the Child–Pugh system [22].

**Table 1:** Patient characteristics

Patient Characteristics	
Mean Age (years)	61 ± 14
Male	n = 42
Female	n = 56
BMI	26.4 ± 5.4 kg/m^2^
Right sided PVE	n = 91
Left sided PVE	n = 7
Additional right sided LVD	n = 10
Underwent subsequent liver resection	n = 82
Tumor entity	
CCC	n = 53
HCC	n = 5
Colorectal liver metastasis	n = 22
Pancreatic adenocarcinoma	n = 2
Neuroendocrine tumors of the gastrointestinal tract	n = 2
Larynx carcinoma	n = 2
Gallbladder carcinoma	n = 3
Benign lesions	n = 9
Previous therapies	n = 45
Prior chemotherapy	n = 38
Prior liver resection	n = 22
Liver cirrhosis (Child A)	n = 2
Liver fibrosis	n = 7
Cholestasis	n = 51
Bilaterally	n = 6
FLR-sided	n = 5
On the side of the embolized lobe (usually with bile-duct stent)	n = 6

### Data acquisition

Imaging was performed no more than four weeks prior to PVE using either magnetic resonance imaging (MRI) or computed tomography (CT). Follow-up imaging was performed six to eight weeks after PVE, again using either MRI or CT. Care was taken to ensure that pre- and post-interventional follow-up was performed using the same imaging modality for each patient. Accordingly, pre- and post-PVE measurements were performed using the same imaging modality in each patient, and comparability between modalities was ensured.

All pre- and post-interventional measurements were performed by a single observer using IntelliSpace Portal software (Philips Healthcare, Best, The Netherlands) and were independently reviewed by two senior radiologists. The following parameters were assessed: maximum diameter [mm] of the portal vein (PV), superior mesenteric vein (SMV), and splenic vein (SV); liver volume [ml], including left and right liver lobes and total liver volume, with particular focus on the future liver remnant (FLR); and splenic volume before and after PVE.

In cases with available MRI using intracellular contrast medium, signal intensities of the left and right liver lobes were measured before and after PVE. Regions of interest (ROIs) with a diameter of 10 mm were placed, ensuring inclusion of liver parenchyma only and exclusion of large vessels.

Post-PVE CT scans were qualitatively assessed for the presence of arterial hyperperfusion of the embolized liver lobe (yes/no). Additional recorded parameters included tumor entity, embolic agent used for PVE (liquid vs. particles and coils), side of PVE (left/right), presence of cholestasis (yes/no), performance of surgical resection (yes/no), hypertrophy time in days (defined as the interval between PVE and preoperative control CT or, in six cases, before in situ split), length of stay in the intensive care unit (ICU) after resection, total postoperative hospital stay, and occurrence of PVE-related complications.

All parameters were analyzed with respect to their influence on FLR growth. Arterial hyperperfusion was defined as arterial-phase parenchymal hyperenhancement of the embolized liver lobe compared with the FLR. The arterial phase was acquired 20 s after contrast medium injection. Cholestasis was defined as dilation of lobar or segmental intrahepatic bile ducts. FLR growth was defined as the increase in volume of the liver lobe remaining in situ after resection, measured on pre- and post-PVE imaging, and expressed as either relative [%] or absolute [ml] growth. In cases of left-sided PVE, the left lobe was embolized and the FLR was located on the right side.

### Portal vein embolization

All PVE procedures were performed using a flat-panel angiography system (Azurion Clarity IQ, Philips Healthcare, Best, The Netherlands). After sonographic identification of portal vein branches and, if required, hepatic veins, sterile conditions were established. Following local anesthesia with lidocaine, a segmental portal vein branch was punctured under sonographic guidance. A 0.014-inch guidewire was introduced, followed by placement of a NEFF set (Cook Medical, Bloomington, Illinois, USA). Subsequently, a 4F reverse-curve catheter (SIM or SOS) was advanced.

**Fig. 2:**
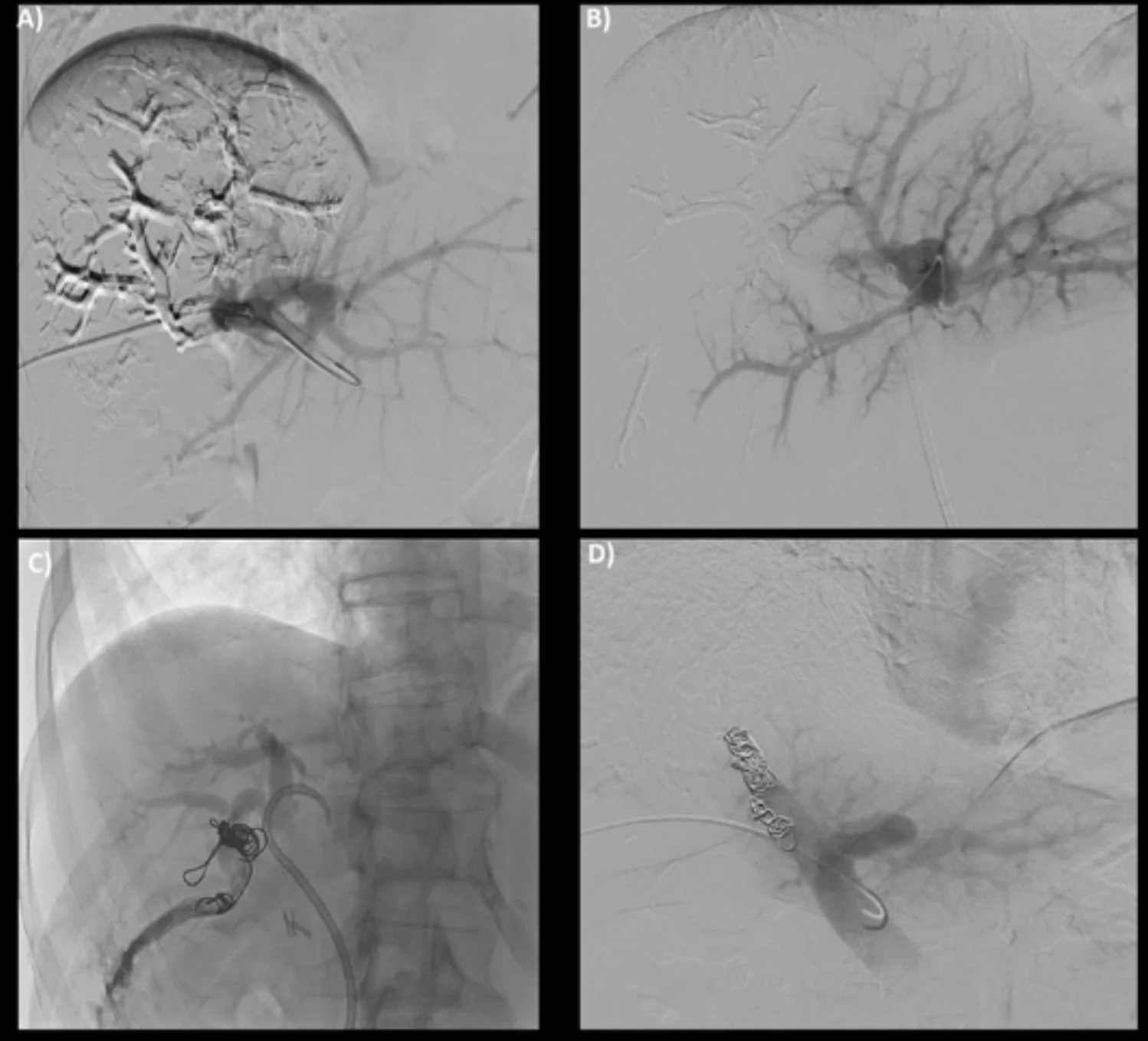
Overview angiographies after portal vein embolization on the right with glue (**A** and **B**) or particles and coils (**C** and **D**)

Overview angiography was performed in posteroanterior and, if necessary, oblique projections. After confirmation of portal vein anatomy, embolization of the right or left portal vein was performed using either particles (500–900 µm) and coils (Azur HydroCoils 0.035, Terumo, Tokyo, Japan) or a liquid embolic agent consisting of Histoacryl (B. Braun, Melsungen, Germany) mixed with Lipiodol (Guerbet, Villepinte, France) in a 1:7 ratio under fluoroscopic guidance (Fig. 2). The choice of embolic materials (particles/coils versus glue) was based on the interventionalist’s preference and material availability, rather than patient-related factors.

After completion of embolization, the puncture tract was sealed using either Gelfoam (Pfizer, New York City, NY, USA) or residual Histoacryl. When hepatic vein occlusion was performed, the corresponding vein was punctured percutaneously via a transhepatic approach under sonographic guidance using the NEFF set. The vein was occluded with a vascular plug (Abbott, Chicago, Illinois, USA) positioned 1–2 cm proximal to the inferior vena cava, followed by embolization with a Histoacryl–Lipiodol mixture (1:2) during catheter withdrawal.

At the end of the procedure, patients were monitored for at least 30 min before transfer to the ward. In the absence of complications, patients were discharged the following day.

### Endpoints

The primary endpoint was relative FLR growth [%] and absolute FLR growth [ml] compared with preinterventional liver lobe volumes. Secondary endpoints included imaging markers such as changes in splenic volume [ml/%], total liver volume, vessel diameters, signal intensities in the hepatobiliary phase, and the presence of arterial hyperperfusion in the embolized liver lobe.

### Statistical analysis

All statistical analyses were performed using SPSS Statistics version 29 (IBM, Armonk, NY, USA). Continuous variables were assessed for normality using the Shapiro–Wilk test and visual inspection of histograms and Q–Q plots. Normally distributed continuous variables are reported as mean ± standard deviation (SD); non-normally distributed variables are reported as median (interquartile range, IQR). Categorical variables are presented as counts and percentages.

For between-group comparisons of continuous variables, the independent-samples t-test was used for normally distributed data, and the Mann–Whitney U test for non-normally distributed data. For comparisons across more than two groups, one-way ANOVA (normal distribution) or the Kruskal–Wallis test (non-normal distribution) was applied. For paired pre-/post-interventional comparisons, the paired t-test was used for normally distributed differences, and the Wilcoxon signed-rank test for non-normally distributed differences. Associations between continuous/ordinal variables were evaluated using Spearman’s rank correlation coefficient (ρ). Two-sided p-values < 0.05 were considered statistically significant.

To identify independent predictors of relative FLR growth (%), a multivariable linear regression model was fitted. The dependent variable was relative FLR growth expressed as percentage change from pre-interventional FLR volume. Independent variables entered simultaneously were LVD (yes/no), hypertrophy time (days), baseline FLR volume (mL), cholestasis category, prior chemotherapy (yes/no), and embolic agent. Model assumptions were assessed using residual diagnostics (normality and homoscedasticity of residuals) and multicollinearity testing (variance inflation factors, VIF). Regression results are reported as unstandardized coefficients (B) with 95% confidence intervals (CI) and p-values.

Derived data supporting the findings of this study are available from the corresponding author (S.E.) upon reasonable request.

## Results

### Study cohort

A total of 122 patients underwent PVE. Twenty-four patients were excluded due to missing imaging data, leaving 98 patients for analysis. Two patients had liver cirrhosis classified as Child–Pugh stage A. Seven patients had liver fibrosis. Five patients had significant hepatic steatosis (> 5%).

Of the 98 patients, 45 had received previous therapies. Thirty-eight patients had undergone chemotherapy prior to inclusion. Twenty-two patients (13 CCC, 8 CRLM, 1 HCC) had undergone liver resection prior to PVE. In eight patients (4 CCC and 4 CRLM), prior resection was performed on the side of the future liver remnant (FLR). In the remaining patients, resection was performed on the side of the subsequent PVE.

PVE was performed on the right side in 91 patients and on the left side in seven patients. Of the 98 patients who underwent PVE, 82 subsequently underwent liver resection. Six patients required an additional in situ split (ALPPS procedure) due to insufficient FLR growth. In the remaining 16 patients, liver resection could not be performed because of extrahepatic tumor manifestations or tumor involvement of the FLR.

### Liver volumes and growth of the future liver remnant

The mean hypertrophy time (interval between PVE and the latest imaging prior to resection) was 38.8 ± 24.2 days (range, 14–63 days). Mean total liver volume before and after PVE was 2002.9 ± 669.2 ml and 1967.7 ± 661.2 ml, respectively, with no significant difference (p = 0.26).

Mean FLR volume increased significantly from 628.9 ± 256.2 before PVE to 979.6 ± 313.8 ml after PVE (p < 0.001). Mean FLR growth was 350.7 ± 275.3 ml, corresponding to a relative increase of 35.6 ± 22.2%.

Mean volume of the embolized liver lobe decreased from 1274.2 ± 470.1 before PVE to 1029.1 ± 435.4 ml after PVE, resulting in a mean volume reduction of 245.1 ± 452.1 ml (19.3 ± 6.1%; p < 0.001). Detailed liver, FLR, and embolized lobe volumes are provided in Table 2.

**Table 2:** Liver, FLR, and embolized lobe volumes

	N	Mean ± SD pre PVE [ml]	Mean ± SD post PVE [ml]	p-value (pre vs. post PVE)
Total liver volume	98	2002.9 ± 669.2	1967.7 ± 661.2	0.26
FLR overall	98	628.9 ± 256	979.6 ± 313.8	** < 0.001**
FLR right liver lobe	7	694.3 ± 114.9	974.6 ± 108.7	** < 0.001**
FLR left liver lobe	91	604.5 ± 287.7	901.3 ± 331.7	** < 0.001**
PVE lobe overall	98	1274.2 ± 470.1	1029.1 ± 435.4	** < 0.001**
PVE right liver lobe	91	1283.1 ± 411.3	1051.2 ± 413.2	** < 0.001**
PVE left liver lobe	7	576.3 ± 249.1	387.1 ± 111.8	** < 0.001**

Significant values are shown in bold

### Liver venous deprivation

In patients who received LVD in addition to PVE (n = 10; all with right-sided PVE using glue and concomitant right hepatic vein occlusion), mean FLR growth was 422 ± 97 ml (61.3 ± 23.1%), which was significantly greater than in patients treated with PVE alone (p < 0.001).

### Influence of the embolic agent

88 patients underwent PVE without LVD. Of these, 56 patients were treated using a liquid embolic agent (glue), and 32 patients were treated with particles and coils. Mean FLR growth after PVE with particles and coils and with a liquid embolic agent was 381.1 ± 287.5 and 394.4 ± 272.5 ml, respectively, without significant (p = 0.11) (Table 3).

**Table 3:** FLR growth

	N	Particles	Liquid embolic agent	p-value (pre vs. post PVE)
FLR-growth overall [ml]	88	381.1 ± 287.5	394.4 ± 274.5	0.11
FLR-growth right liver lobe [ml]	7	294.9 ± 253.1	271.3 ± 272.1	0.13
FLR-growth left liver lobe [ml]	81	460.8 ± 217.7	447.1 ± 211.6	0.1

### Arterial hyperperfusion and liver signal intensity on post-interventional MRI

**Fig. 3:**
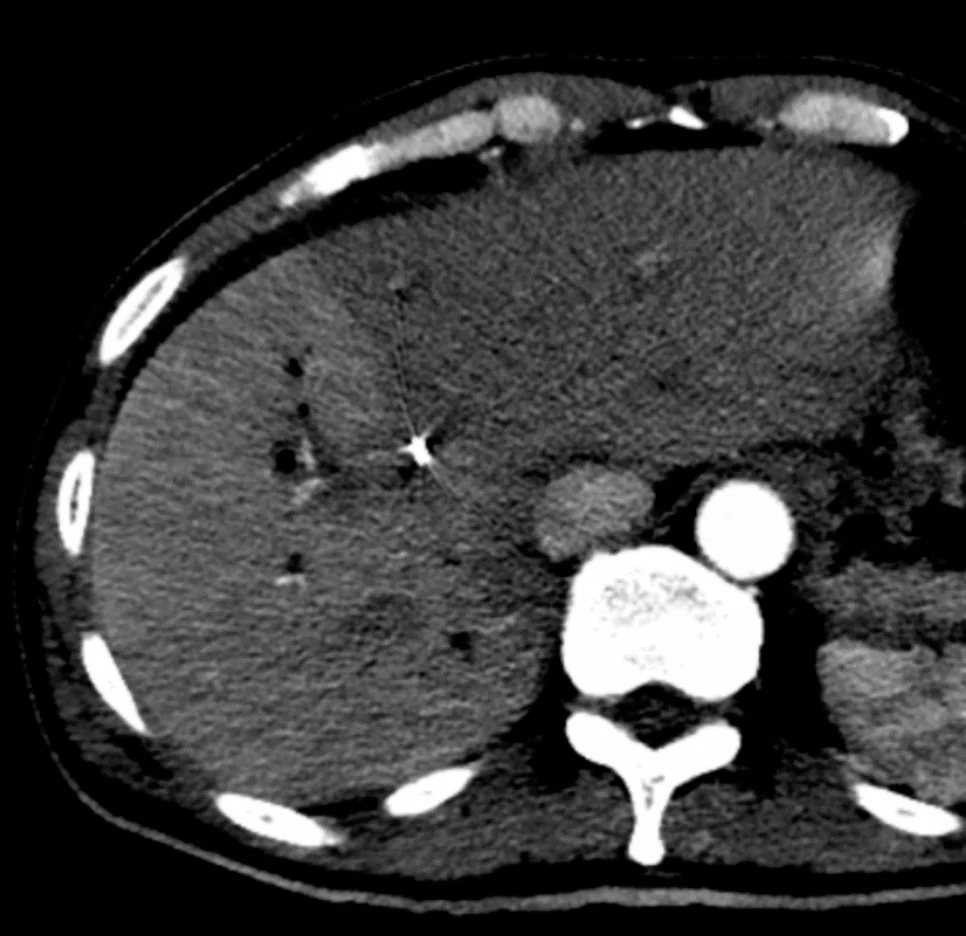
Computed tomographic imaging of the arterial hyperperfusion of the right lobe of the liver after right portal vein embolization

A moderate correlation was observed between the presence of arterial hyperperfusion of the embolized liver lobe on post-interventional imaging (Fig. 3) and FLR growth (R = 0.39; p = 0.036).

In 37 cases, pre- and post-PVE MRI examinations were performed using a liver-specific contrast agent. There were no significant differences in mean signal intensities in the hepatobiliary phase between the FLR and the embolized lobe before and after PVE (FLR: 140 ± 102 vs. 136 ± 94 AU; embolized lobe: 133 ± 94 vs. 129 ± 96 arbitrary units; p = 0.061 for all comparisons).

### Influence of liver-related factors

Two patients had liver cirrhosis classified as Child–Pugh stage A, and seven patients had mild hepatic fibrosis. Neither condition had a statistically significant impact on FLR growth (p = 0.076); however, a trend toward reduced FLR growth was observed in patients with cirrhosis (Supplementary Fig. 1).

In patients with bilateral cholestasis (n = 6) or cholestasis affecting the FLR (n = 5), FLR growth was significantly lower than in patients without cholestasis (p < 0.001). In contrast, patients with isolated cholestasis of the embolized liver lobe (n = 40), typically with bile duct stenting on the FLR side, exhibited significantly greater FLR growth than patients without cholestasis (p = 0.038) (Table 4). None of the embolized liver lobes were drained. No cholangitis was observed.

**Table 4:** FLR growth and cholestasis

	No cholestasis	Cholestasis bilaterally or on FLR-side	Cholestasis on the embolized liver lobe	p-value (no cholestasis vs. Bilaterally or on FLR-side)	p-value (no cholestasis vs. On embolized side)
FLR-growth overall [ml]	389.7 ± 275.0	173.8 ± 113.7	401.7 ± 291.7	** < 0.001**	**0.038**
FLR-growth right liver lobe [ml]	298.3 ± 245.7	201.1 ± 93.3	321.4 ± 211.8	** < 0.001**	**0.041**
FLR-growth left liver lobe [ml]	465.3 ± 219.3	191.1 ± 136.8	497.2 ± 154.8	** < 0.001**	**0.027**

Significant values are shown in bold

No significant association was found between tumor entity and FLR growth (p = 0.11).

### Splenic volume and venous diameters

Splenic volume increased significantly from 271.3 ± 118.5 before PVE to 311.9 ± 146.5 ml after PVE (p < 0.001). No significant differences were observed in the diameters of the portal vein (p = 0.09), splenic vein (p = 0.06), or superior mesenteric vein before and after PVE (p = 0.08) respectively). Correlations between venous diameters and FLR growth were weak (R = 0.02–0.18). Relative splenic growth showed a weak but statistically significant positive correlation with relative FLR growth (Spearman’s ρ = 0.24, p = 0.03).

### Length of stay in the intensive care unit and hospital after liver resection

The mean length of stay in the ICU after liver resection was 4 ± 3 days, and the mean total hospital stay was 24 ± 16 days (range, 8–40 days). No significant associations were found between length of stay and liver volumes, FLR growth, embolic agent, performance of LVD, presence of cholestasis, venous diameters, or splenic volume (p = 0.09, p = 0.06, p = 0.08, respectively). Correlations between venous diameters and FLR growth were weak (R = 0.02–0.18).

### Complications

Two patients developed biliary leakage from the puncture site after PVE, requiring drain placement. One patient developed a subcapsular hematoma that resolved without further intervention. One patient experienced complete portal vein thrombosis, which was successfully recanalized on the FLR side during subsequent resection.

### Multivariable regression

In the multivariable model (Table 5), the overall regression was significant (F = 5.2, p < 0.001) and explained a substantial proportion of variance in relative FLR growth (R^2^ = 0.40, adjusted R^2^ = 0.36). LVD was independently associated with higher relative FLR growth (B =  + 22.0%, 95% CI + 10.5; + 33.5, p = 0.001). Hypertrophy time was also positively associated with relative FLR growth (per 10 days: B =  + 5.2%, 95% CI + 1.8; + 8.6, p = 0.003), whereas baseline FLR volume showed an inverse association (per 100 mL: B =  − 2.6%, 95% CI − 4.8; − 0.4, p = 0.021). Compared with no cholestasis, cholestasis confined to the embolized lobe was associated with higher relative FLR growth (B =  + 8.7%, 95% CI + 0.3; + 17.1, p = 0.043), while cholestasis involving the FLR and/or bilateral cholestasis was negatively associated (B =  − 17.5%, 95% CI − 35.8; + 0.8, p = 0.061). Prior chemotherapy showed a non-significant trend toward lower relative FLR growth (B =  − 6.2%, 95% CI − 13.5; + 1.1, p = 0.095), and embolic agent (particles/coils vs glue) was not associated with relative FLR growth (B =  + 1.1%, 95% CI − 5.8; + 8.0, p = 0.75).

**Table 5:** Data are presented as unstandardized regression coefficients (B) with 95% confidence intervals (CI)

Variable	B	95% CI	p value
LVD (yes vs no)	+ 22.0	+ 10.5 to + 33.5	0.001
Hypertrophy time (per 10 days)	+ 5.2	+ 1.8 to + 8.6	0.003
Baseline FLR volume (per 100 mL)	− 2.6	− 4.8 to − 0.4	0.021
Cholestasis confined to embolized lobe (vs no cholestasis)	+ 8.7	+ 0.3 to + 17.1	0.043
Cholestasis involving FLR and/or bilateral cholestasis (vs no cholestasis)	− 17.5	− 35.8 to + 0.8	0.061
Prior chemotherapy (yes vs no)	− 6.2	− 13.5 to + 1.1	0.095
Embolic agent (particles/coils vs glue)	+ 1.1	− 5.8 to + 8.0	0.750

The dependent variable was relative future liver remnant (FLR) growth (%). Variables entered simultaneously into the model were liver venous deprivation (LVD), hypertrophy time, baseline FLR volume, cholestasis category, prior chemotherapy, and embolic agent. The reference category for cholestasis was no cholestasis; for embolic agent, glue was the reference category. Overall model performance: F = 5.2, p < 0.001, R^2^ = 0.40, adjusted R^2^ = 0.36

## Discussion

In this single-center retrospective cohort of 98 patients undergoing portal vein embolization (PVE) prior to major hepatectomy, we identified several imaging markers associated with future liver remnant (FLR) hypertrophy. Mean FLR growth was 35.6 ± 22.2%, consistent with previously reported values [23]. In our small cohort concomitant liver venous deprivation (LVD) was associated with significantly greater FLR hypertrophy compared with PVE alone (61.3 ± 23.1%), supporting the previously described regenerative advantage of combined inflow–outflow deprivation [9, 24]. Previously unobserved associations were identified. An association between the presence of arterial hyperperfusion in the embolized liver lobe post-PVE and greater FLR hypertrophy was observed. In addition, isolated cholestasis limited to the embolized lobe was associated with enhanced FLR growth, whereas cholestasis involving the FLR was associated with impaired hypertrophy. A significant increase in splenic volume following PVE was documented. No significant difference in FLR growth was observed between liquid embolic agents and particle/coil-based embolization techniques, in contrast to some prior reports [6, 7]. Collectively, arterial hyperperfusion, ipsilateral cholestasis, and post-PVE splenic enlargement emerge as candidate imaging markers for individualized risk stratification and patient selection prior to liver resection, pending further validation.

The previously unreported association between arterial hyperperfusion of the embolized liver lobe and increased FLR hypertrophy may reflect compensatory arterial inflow following portal vein occlusion, but alternative biological and hemodynamic mechanisms are likely involved. Importantly, substantial FLR hypertrophy was also observed in the absence of arterial hyperperfusion, emphasizing that this imaging feature should be interpreted as a favorable prognostic marker rather than a prerequisite for successful regeneration.

Interestingly, we observed a significant association between increased FLR hypertrophy and isolated cholestasis confined to the embolized liver lobe. This finding has previously been reported only as an incidental observation by Chang et al. [25]. Cholestasis is a recognized atrophic stimulus, and its cellular mechanisms have been described in detail by Huang et al. [26]. Accordingly, cholestasis affecting the FLR was associated with impaired hypertrophy in our cohort. In contrast, when restricted to the embolized liver lobe, cholestasis appears to be associated with compensatory FLR hypertrophy. Whether this paradoxical association could be therapeutically exploited—for example through modulation of biliary drainage—remains speculative and, given the limited sample sizes in the respective subgroups, should be regarded as hypothesis-generating.

Another notable observation was the development of new-onset splenomegaly on preoperative imaging following PVE. While this finding has not been previously described, its clinical relevance remains uncertain. Given the established role of the spleen in portal hypertension as a regulator of splanchnic blood flow [27], splenic enlargement may reflect an acute increase in portal venous pressure after embolization, with the spleen acting as a compensatory reservoir. The observed weak positive correlation between relative splenic growth and relative FLR growth further supports the hypothesis that splenic enlargement after PVE reflects the magnitude of hemodynamic adaptation to portal flow redistribution and may therefore represent an indirect marker of regenerative activity in the FLR. However, given the limited strength of the association, this finding should be interpreted cautiously and requires validation in prospective studies. The absence of venous collateral formation in most patients may be attributable to preserved hepatic compliance and regenerative capacity of the FLR, in contrast to cirrhotic livers with chronic portal hypertension. Direct measurements in clinical studies have shown that portal venous pressure increases following PVE. For example, in a cohort of patients undergoing PVE prior to major hepatectomy, median portal venous pressure increased from 15 (range 9–24) before PVE to 19 mm Hg (range 10–27) after PVE. Similarly, the hepatic venous pressure gradient increased from a median of 5 (range 0–12) to 8 mm Hg (range 0–14) post-PVE. These hemodynamic changes have been shown to correlate with the degree of future liver remnant hypertrophy, with higher post-PVE pressure gradients predicting impaired hypertrophy response [28]. In this sense, splenic enlargement could mirror the magnitude of the pressure response that—according to the cited study—also relates to the subsequent hypertrophy performance of the FLR.

Although not statistically significant due to limited sample size, the observed trend toward reduced FLR hypertrophy in patients with cirrhosis or advanced fibrosis aligns with the established reduction in regenerative capacity of chronically diseased liver parenchyma [29]. This finding further emphasizes the importance of tailoring preoperative hypertrophy-inducing strategies according to baseline liver quality, functional reserve, and prior oncologic treatments.

Approximately 15% of patients did not proceed to surgical resection, exclusively due to interval tumor progression. Comparable non-resection rates of up to 20% have been reported in the literature^1^. Contributing factors include insufficient FLR hypertrophy, hyperbilirubinemia, prior systemic chemotherapy, and oncologic progression during the hypertrophy interval [30, 31]. However, baseline differences between resected and non-resected patients cannot be fully excluded due to the retrospective nature of the study. However, these observations raise the question of whether a “test-of-time” strategy associated with slower hypertrophy—such as that observed following yttrium-90 radioembolization—may facilitate improved oncologic selection rather than faster volumetric gain [32]. Nevertheless, this approach remains insufficiently validated and carries inherent risks, including tumor progression and abscess formation within the embolized liver lobe [33], which may preclude curative resection. Together, these considerations highlight the need for reliable predictors of both regenerative capacity and oncologic outcome in patients undergoing hypertrophy-inducing procedures.

The overall complication rate in our cohort was 3%, including two biliary leaks and one subcapsular hematoma that resolved without further intervention. This rate is consistent with the expected complication rate of approximately 2.5% reported in the CIRSE guidelines [34], which list capsular hematoma, arteriovenous shunts, pseudoaneurysms, cholangitis, and thrombosis as the most common procedure-related complications.

Several limitations should be acknowledged. The retrospective, monocentric design and single-reader image assessment limit generalizability and preclude causal inference. In addition, fewer than 10% of patients underwent combined hepatic vein deprivation, restricting the statistical power of subgroup analyses. The relatively small number of patients undergoing LVD (n = 10) and left-sided PVE (n = 7) limits the robustness of subgroup analyses. The use of both CT and MRI for volumetric assessment may introduce measurement variability. But care was taken to ensure that pre- and post-interventional follow-up was performed using the same imaging modality for each patient. Relevant confounders such as liver stiffness, functional reserve, portal pressure, and tumor biology are known to influence both FLR regeneration and oncologic outcomes. These parameters should be incorporated into future prospective studies to refine patient selection and procedural decision-making.

In conclusion, PVE is a safe and effective technique for inducing FLR hypertrophy. Imaging features identified in this study—including arterial hyperperfusion, ipsilateral cholestasis, and splenic enlargement—are associated with regenerative response and may provide early prognostic information, whereas underlying parenchymal disease appears to limit hypertrophic capacity. These findings may help refine patient selection and warrant prospective validation, although multicenter validation is required before these parameters can be incorporated into clinical decision algorithms.

## Supplementary Information

Below is the link to the electronic supplementary material.

Supplementary file1 (DOCX 31 KB)

## Data Availability

The datasets used and/or analyzed during the current study are available from the corresponding author on reasonable request.

## References

[1] Cassese, G. *et al.* Portal vein embolization failure: Current strategies and future perspectives to improve liver hypertrophy before major oncological liver resection. *World J. Gastrointest. Oncol.* **14**, 2088–2096 (2022). 10.4251/wjgo.v14.i11.2088 PMC9694272 36438704

[2] Sivakumar, I. & Liddell, R. P. A review of portal vein embolization prior to hepatic resection. *J. Surg. Oncol.* **130**, 435–442 (2024). 10.1002/jso.27738 39402904

[3] Huang, Y. *et al.* Preoperative Portal Vein Embolization for Liver Resection: An updated meta-analysis. *J. Cancer* **12**, 1770–1778 (2021). 10.7150/jca.50371 PMC7890316 33613766

[4] Werey, F. *et al.* Right portal vein ligation is still relevant for left hemi-liver hypertrophy: results of a comparative study using a propensity score between right portal vein ligation and embolization. *Langenbecks Arch. Surg.* **409**, 25 (2023). 10.1007/s00423-023-03213-8 38158401

[5] Rassam, F. *et al.* Functional and volumetric assessment of liver segments after portal vein embolization: Differences in hypertrophy response. *Surgery* **165**, 686–695 (2019). 10.1016/j.surg.2018.11.004 30573191

[6] Guiu, B. *et al.* Portal vein embolization before right hepatectomy: improved results using n-butyl-cyanoacrylate compared to microparticles plus coils. *Cardiovasc. Intervent. Radiol.* **36**, 1306–1312 (2013). 10.1007/s00270-013-0565-7 23361117

[7] Ali, A., Ahle, M., Björnsson, B. & Sandström, P. Portal vein embolization with N-butyl cyanoacrylate glue is superior to other materials: a systematic review and meta-analysis. *Eur. Radiol.* **31**, 5464–5478 (2021). 10.1007/s00330-020-07685-w 33501598

[8] 9.Carling, U., Røsok, B., Berger, S., Fretland, Å. A. & Dorenberg, E. Portal Vein Embolization Using N-Butyl Cyanoacrylate-Glue: What Impact Does a Central Vascular Plug Have? *Cardiovasc. Intervent. Radiol.* **45**, 450–458 (2022). 10.1007/s00270-021-03014-w PMC8940786 34907454

[9] 10.Heil, J. & Schadde, E. Simultaneous portal and hepatic vein embolization before major liver resection. *Langenbecks Arch. Surg.* **406**, 1295–1305 (2021). 10.1007/s00423-020-01960-6 PMC8370912 32839889

[10] 11.Gavriilidis, P., Marangoni, G., Ahmad, J. & Azoulay, D. Simultaneous portal and hepatic vein embolization is better than portal embolization or ALPPS for hypertrophy of future liver remnant before major hepatectomy: A systematic review and network meta-analysis. *Hepatobiliary Pancreat. Dis. Int. HBPD INT* **22**, 221–227 (2023). 10.1016/j.hbpd.2022.08.013 36100542

[11] 12.Yang, L. *et al.* Comparison of liver venous deprivation with portal vein embolization alone in patients undergoing major liver resection: a systematic review and meta-analysis. *HPB* **26**, 1329–1338 (2024). 10.1016/j.hpb.2024.07.409 39054212

[12] 13.Gadani, S. *et al.* Liver Vein Deprivation versus Portal Vein Embolization: Retrospective Review of Safety and Effectiveness. *J. Vasc. Interv. Radiol.* **36**, 31-39.e2 (2025). 10.1016/j.jvir.2024.09.025 39389233

[13] 14.Böning, G. *et al.* Liver Venous Deprivation (LVD) Versus Portal Vein Embolization (PVE) Alone Prior to Extended Hepatectomy: A Matched Pair Analysis. *Cardiovasc. Intervent. Radiol.* **45**, 950–957 (2022). 10.1007/s00270-022-03107-0 PMC9226084 35314879

[14] 15.Li, A. Y. *et al.* Postoperative hepatic insufficiency despite preoperative portal vein embolization: Not just about the volumetrics. *Surgery* **182**, 109345 (2025). 10.1016/j.surg.2025.109345 40157125

[15] 16.Barth, B. K., Fischer, M. A., Kambakamba, P., Lesurtel, M. & Reiner, C. S. Liver-fat and liver-function indices derived from Gd-EOB-DTPA-enhanced liver MRI for prediction of future liver remnant growth after portal vein occlusion. *Eur. J. Radiol.* **85**, 843–849 (2016). 10.1016/j.ejrad.2016.02.008 26971433

[16] 17.Araki, K. *et al.* Functional remnant liver volumetry using Gd-EOB-DTPA-enhanced magnetic resonance imaging (MRI) predicts post-hepatectomy liver failure in resection of more than one segment. *HPB* **22**, 318–327 (2020). 10.1016/j.hpb.2019.08.002 31477460

[17] 18.Bartholomä, W. C. *et al.* Magnetic resonance-derived hepatic uptake index improves the identification of patients at risk of severe post-hepatectomy liver failure. *Br. J. Surg.* **112**, znaf103 (2025). 10.1093/bjs/znaf103 PMC12120443 40439010

[18] 19.Soykan, E. A. *et al.* Predictive Factors for Hypertrophy of the Future Liver Remnant After Portal Vein Embolization: A Systematic Review. *Cardiovasc. Intervent. Radiol.* **44**, 1355–1366 (2021). 10.1007/s00270-021-02877-3 PMC8382618 34142192

[19] 20.Kohno, S. *et al.* Portal Vein Embolization: Radiological Findings Predicting Future Liver Remnant Hypertrophy. *AJR Am. J. Roentgenol.* **214**, 687–693 (2020). 10.2214/AJR.19.21440 31642696

[20] 21.Takahashi, E. A., Fleming, C. J. & Andrews, J. C. Future Liver Remnant Hypertrophy after Portal Vein Embolization Is Inversely Correlated with Intrahepatic Tumor Burden. *J. Vasc. Interv. Radiol.* **30**, 435–439 (2019). 10.1016/j.jvir.2018.10.014 30819489

[21] 23.Eddowes, P. J. *et al.* Accuracy of FibroScan Controlled Attenuation Parameter and Liver Stiffness Measurement in Assessing Steatosis and Fibrosis in Patients With Nonalcoholic Fatty Liver Disease. *Gastroenterology* **156**, 1717–1730 (2019). 10.1053/j.gastro.2019.01.042 30689971

[22] 24.Infante-Rivard, C., Esnaola, S. & Villeneuve, J.-P. Clinical and statistical validity of conventional prognostic factors in predicting short-term survival among cirrhotics. *Hepatology* **7**, 660–664 (1987). 10.1002/hep.1840070408 3610046

[23] 25.Chaouch, M. A. *et al.* A systematic review and meta-analysis of liver venous deprivation versus portal vein embolization before hepatectomy: future liver volume, postoperative outcomes, and oncological safety. *Front. Med.* **10**, 1334661 (2024). 10.3389/fmed.2023.1334661 PMC10806199 38269320

[24] 26.Al Taweel, B. *et al.* Assessment of Segmentary Hypertrophy of Future Remnant Liver after Liver Venous Deprivation: A Single-Center Study. *Cancers* **16**, 1982 (2024). 10.3390/cancers16111982 PMC11171007 38893103

[25] 27.Chang, X. *et al.* Influence of cholestasis on portal vein embolization-induced hypertrophy of the future liver remnant. *Langenbecks Arch. Surg.* **408**, 54 (2023). 10.1007/s00423-023-02784-w PMC9867667 36680689

[26] 28.Huang, K. *et al.* Bile acids attenuate hepatic inflammation during ischemia/reperfusion injury. *JHEP Rep.* **6**, 101101 (2024). 10.1016/j.jhepr.2024.101101 PMC11292370 39091991

[27] The Role of the Spleen in Portal Hypertension - PubMed. https://pubmed.ncbi.nlm.nih.gov/36908126/.

[28] 30.Mohkam, K. *et al.* Hepatic venous pressure gradient after portal vein embolization: An accurate predictor of future liver remnant hypertrophy. *Surgery* **164**, 227–232 (2018). 10.1016/j.surg.2018.03.014 29753461

[29] 31.Primavesi, F. *et al.* E-AHPBA–ESSO–ESSR Innsbruck consensus guidelines for preoperative liver function assessment before hepatectomy. *Br. J. Surg.* **110**, 1331–1347 (2023). 10.1093/bjs/znad233 PMC10480040 37572099

[30] 32.Abdelrafee, A. *et al.* Predictors of unresectability after portal vein embolization for centrally located cholangiocarcinoma. *Surgery* **168**, 287–296 (2020). 10.1016/j.surg.2020.03.005 32340719

[31] 33.Hoekstra, L. T. *et al.* Tumor Progression After Preoperative Portal Vein Embolization. *Ann. Surg.* **256**, 812–818 (2012). 10.1097/SLA.0b013e3182733f09 23095626

[32] 34.Birgin, E. *et al.* Contralateral Liver Hypertrophy and Oncological Outcome Following Radioembolization with 90Y-Microspheres: A Systematic Review. *Cancers* **12**, 294 (2020). 10.3390/cancers12020294 PMC7072354 32012709

[33] 35.Huisman, F., Cieslak, K. P., van Lienden, K. P., Bennink, R. J. & van Gulik, T. M. Liver related complications in unresectable disease after portal vein embolization. *Hepatobiliary Surg. Nutr.* **6**, 379–386 (2017). 10.21037/hbsn.2017.02.03 PMC5756764 29312972

[34] 36.Bilhim, T., Böning, G., Guiu, B., Luz, J. H. & Denys, A. CIRSE Standards of Practice on Portal Vein Embolization and Double Vein Embolization/Liver Venous Deprivation. *Cardiovasc. Intervent. Radiol.* **47**, 1025–1036 (2024). 10.1007/s00270-024-03743-8 PMC11303578 38884781

