# Supplementary material for: Imaging biomarkers of liver hypertrophy after portal vein embolization in patients with liver tumors: cholestasis, arterial hyperperfusion, and splenic response
Source: Abdom Radiol (NY). 2026 May 12;51(11):5827–37. doi: 10.1007/s00261-026-05540-y (PMC13672558; doi:10.1007/s00261-026-05540-y)
Supplement: Supplementary file 1 [file 261_2026_5540_MOESM1_ESM.docx]

Supplemental Figure 1

Boxplot diagram showing a trend towards poorer growth of the Future Liver Remnant when cirrhosis is present, although the difference is not statistically significant.
